# Auraptene and Other Prenyloxyphenylpropanoids Suppress Microglial Activation and Dopaminergic Neuronal Cell Death in a Lipopolysaccharide-Induced Model of Parkinson’s Disease

**DOI:** 10.3390/ijms17101716

**Published:** 2016-10-17

**Authors:** Satoshi Okuyama, Tomoki Semba, Nobuki Toyoda, Francesco Epifano, Salvatore Genovese, Serena Fiorito, Vito Alessandro Taddeo, Atsushi Sawamoto, Mitsunari Nakajima, Yoshiko Furukawa

**Affiliations:** 1 Department of Pharmaceutical Pharmacology, College of Pharmaceutical Sciences, Matsuyama University, 4-2 Bunkyo-cho, Matsuyama, Ehime 790-8578, Japan; mu.yakuri.001@gmail.com (T.S.); kowagari77@yahoo.co.jp (N.T.); 46140018@cc.matsuyama-u.ac.jp (A.S.); mnakajim@cc.matsuyama-u.ac.jp (M.N.); furukawa@cc.matsuyama-u.ac.jp (Y.F.); 2 Department of Pharmacy, University “G. D’Annunzio”, Chieti-Pescara Via dei Vestini 31, Chieti Scalo 66100, Italy; fepifano@unich.it (F.E.); s.genovese@unich.it (S.G.); serena.fiorito@unich.it (S.F.); vito.taddeo@unich.it (V.A.T.)

**Keywords:** auraptene, 7-isopentenyloxycoumarin, 4′-geranyloxyferulic acid, prenyloxyphenylpropanoids, substantia nigra, Parkinson’s disease, anti-inflammation, microglia, neuroprotection

## Abstract

In patients with Parkinson’s disease (PD), hyperactivated inflammation in the brain, particularly microglial hyperactivation in the substantia nigra (SN), is reported to be one of the triggers for the delayed loss of dopaminergic neurons and sequential motor functional impairments. We previously reported that (1) auraptene (AUR), a natural prenyloxycoumain, suppressed inflammatory responses including the hyperactivation of microglia in the ischemic brain and inflamed brain, thereby inhibiting neuronal cell death; (2) 7-isopentenyloxycoumarin (7-IP), another natural prenyloxycoumain, exerted anti-inflammatory and neuroprotective effects against excitotoxicity; and (3) 4′-geranyloxyferulic acid (GOFA), a natural prenyloxycinnamic acid, also exerted anti-inflammatory effects. In the present study, using an intranigral lipopolysaccharide (LPS)-induced PD-like mouse model, we investigated whether AUR, 7-IP, and GOFA suppress microglial activation and protect against dopaminergic neuronal cell death in the SN. We successfully showed that these prenyloxyphenylpropanoids exhibited these prospective abilities, suggesting the potential of these compounds as neuroprotective agents for patients with PD.

## 1. Introduction

Prenyloxyphenylpropanoids are *C*-prenylated (isopentenyl-, geranyl-, and farnesyl-) derivatives of phenylpropanoids (including coumarins, cinnamic acid, and benzoic acid). To date, several hundred related compounds have been isolated from plants (mainly Rutaceae, Apiaceae, and Compositae) containing edible plants and fruits, and are regarded as the secondary metabolites of phenylpropanoids. In the last few decades, numerous extensive studies have revealed that these natural prenyloxyphenylpropanoids are important bioactive substances because they exhibit various important activities, such as anti-cancer, anti-inflammatory, and anti-microbial activities [1].

**Figure 1:**
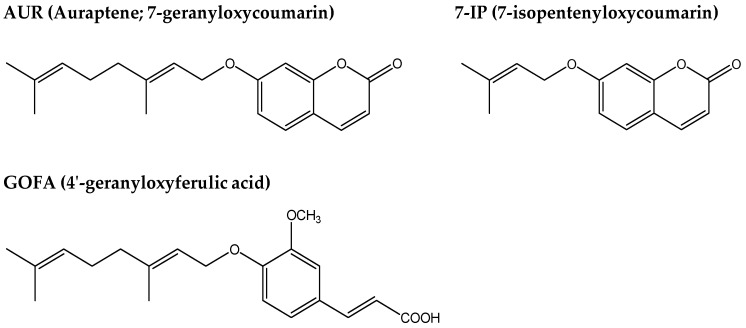
Structures of 7-geranyloxycoumarin (auraptene; AUR), 7-isopentenyloxycoumarin (7-IP), and 4′-geranyloxyferulic acid (GOFA).

Similar to other prenyloxyphenylpropanoids, auraptene (AUR), a 7-geranyloxylated coumarin (Figure 1), exhibits potent anti-cancer and anti-inflammatory activities [2,3,4], and also scavenges free radicals in leukocytes [5] and the ischemic brain [6]. Epifano and co-workers showed that AUR exerted in vitro neuroprotective effects against *N*-methyl-d-aspartate (NMDA)-induced excitotoxicity in a mixed cortical cell culture [7] and in vivo neuroprotective activity in a maximal electroshock-induced seizure mouse model [8]. Furthermore, Okuyama and co-workers recently reported that AUR exerts anti-inflammatory effects not only in peripheral tissues, but also in the brain because it suppressed inflammatory responses in the ischemic brain [9,10] and ameliorated lipopolysaccharide (LPS)-induced inflammation in the mouse brain [11]. In the present study, we focused on the suppressive effects of AUR on the activation of microglia in the brain because recent studies reported that the pathogenesis of Parkinson’s disease (PD) is closely associated with inflammation [12], mainly activated microglia [13].

Several neurotoxic compounds including 6-hydroxydopamine (6-OHDA) and 1-methyl-4-phenyl-1,2,3,6-tetrahydropyridine (MPTP) were used to prepare PD model animals. 6-OHDA induces oxidative stress and mitochondrial dysfunction by regulating mitochondrial dynamics [14]. MPTP is metabolized to MPP+, which produces reactive oxygen species (ROS) and disrupts the mitochondrial membrane potential [15]. In the present study, we used LPS, an endotoxin of Gram-negative bacteria, as a neurotoxin for the preparation of PD model mice because its infusion into the SN has been reported to activate microglia in this region, resulting in the generation of proinflammatory cytokines and neurotoxic factors [16,17,18,19,20].

Together with AUR, we herein examined the effects of other prenyloxyphenylpropanoids such as 7-isopentenyloxycoumarin (7-IP) and 3-(4′-geranyloxy-3′-methoxyphenyl)-2-trans propenoic acid (4′-geranyloxyferulic acid; GOFA) (Figure 1) in the PD-like model mice generated. 7-IP, a 7-prenyloxycoumain, exerted anti-inflammatory and anti-cancer effects [21,22], in vitro neuroprotective effects against NMDA-induced excitotoxicity in a mixed cortical cell culture [7], and in vivo neuroprotective activity in a maximal electroshock-induced seizure mouse model, similar to AUR [8]. GOFA, a prenyloxycinnamic acid, has been identified as an inhibitor of cyclooxygenase (COX)-2 and inducible nitric oxide synthase (iNOS) for cancer prevention [23]. Epifano and co-workers showed that it exhibited anti-inflammatory activity in isolated human monocytes stimulated with LPS [24] as well as anti-cancer activity [25,26].

## 2. Results

### 2.1. Effects of AUR, 7-IP, and GOFA on the LPS-Induced Sickness Response

**Figure 2:**
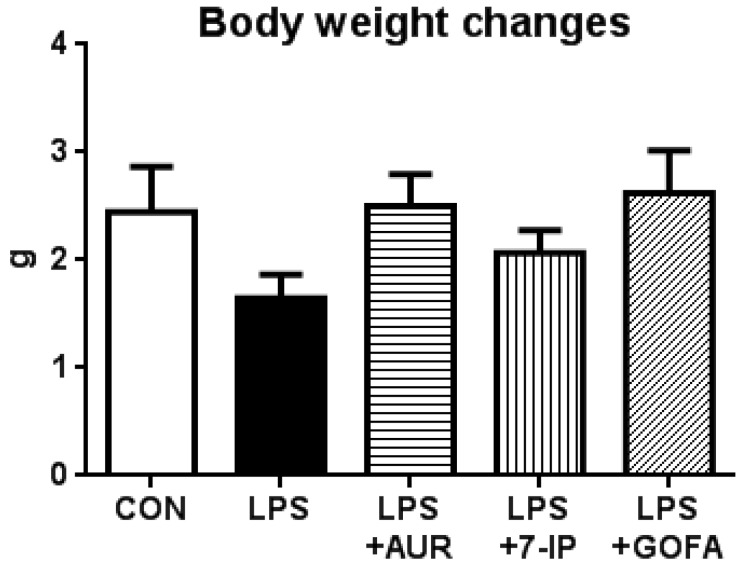
Effects of AUR, 7-IP, and GOFA on lipopolysaccharide (LPS)-induced body weight loss. The control group was defined as CON. Values (means ± SEM) are changes in body weight from day 1 to that on day 22.

The LPS-induced sickness response is reflected by changes in body weight gain [20]. Therefore, we measured body weight on days 1 and 22 (one day after the daily administration of each sample for 21 days; 21 days after a single intranigral injection of LPS). As shown in Figure 2, body weight gain during the experimental period by mice in the LPS group (1.64 ± 0.22 g) was markedly lower than that by mice in the control group (CON) (2.44 ± 0.42 g). Body weight gains by mice in the LPS + AUR and LPS + GOFA groups were 2.50 ± 0.30 g and 2.62 ± 0.39 g, respectively, indicating that AUR and GOFA both have the ability to suppress LPS-induced body weight losses; however, no significant differences were observed among these groups. Body weight gain by mice in the LPS + 7-IP group was 2.07 ± 0.21 g, indicating that 7-IP also exhibits a weak ability to suppress LPS-induced body weight loss. These results suggest that prenyloxycoumains exhibit a weak ability to ameliorate the LPS-induced sickness response.

### 2.2. Effects of AUR, 7-IP, and GOFA on LPS-Induced Microglial Hyperactivation in the SN

**Figure 3:**
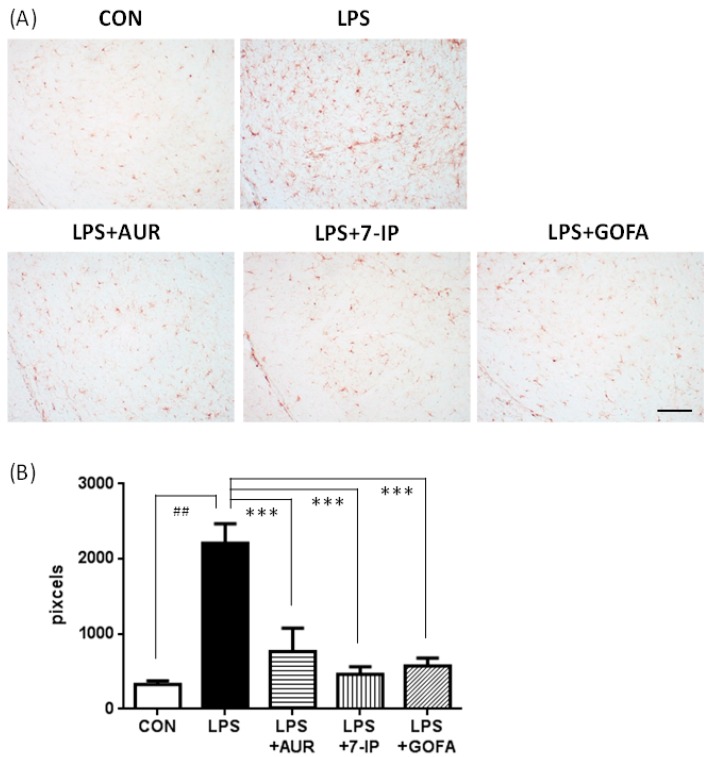
Effects of AUR, 7-IP, and GOFA on LPS-induced microglial hyperactivation in the substantia nigra. (**A**) Representative micrographs of IBA1-positive cells on day 22 in the indicated groups. Scale bar = 100 μm; (**B**) Total IBA1-positive cells signal densities. Values are the means ± SEM. Significant differences were observed between control (CON) and LPS (^##^ *p* < 0.01) and between LPS and sample treatment groups (*** *p* < 0.001).

Microglial cells were stained with an antibody against IBA1, a well-known marker of microglia/macrophages. In the CON group, only a few IBA1-positive cells were observed as the inactivated form (smaller cell bodies; ramified microglia) (Figure 3A). In the LPS group, the shape of IBA1-positive cells changed to the activated form (larger cell bodies and short ramifications; ameboid microglia) (Figure 3A). When the area of IBA1-positive cells was measured, LPS group was approximately 6.7-fold that of the CON group (Figure 3B; ^##^ *p* < 0.01). In the LPS + AUR, LPS + 7-IP, and LPS + GOFA groups, the shape of most IBA1-positive cells returned to the inactivated ramified form (Figure 3A). As shown in Figure 3B, the area of these cells in the LPS + AUR group was approximately 39.7% that of the LPS group (*** *p* < 0.001), that in the LPS + 7-IP group was approximately 19.1% that in the LPS group (*** *p* < 0.001), and that in the LPS + GOFA group was approximately 26.1% that in the LPS group (*** *p* < 0.001). These results indicate that AUR, 7-IP, and GOFA exhibit potent abilities to suppress LPS-induced microglial hyperactivation in the SN.

### 2.3. Effects of AUR, 7-IP, and GOFA on the LPS-Induced Activation of Astrocytes in the SN

**Figure 4:**
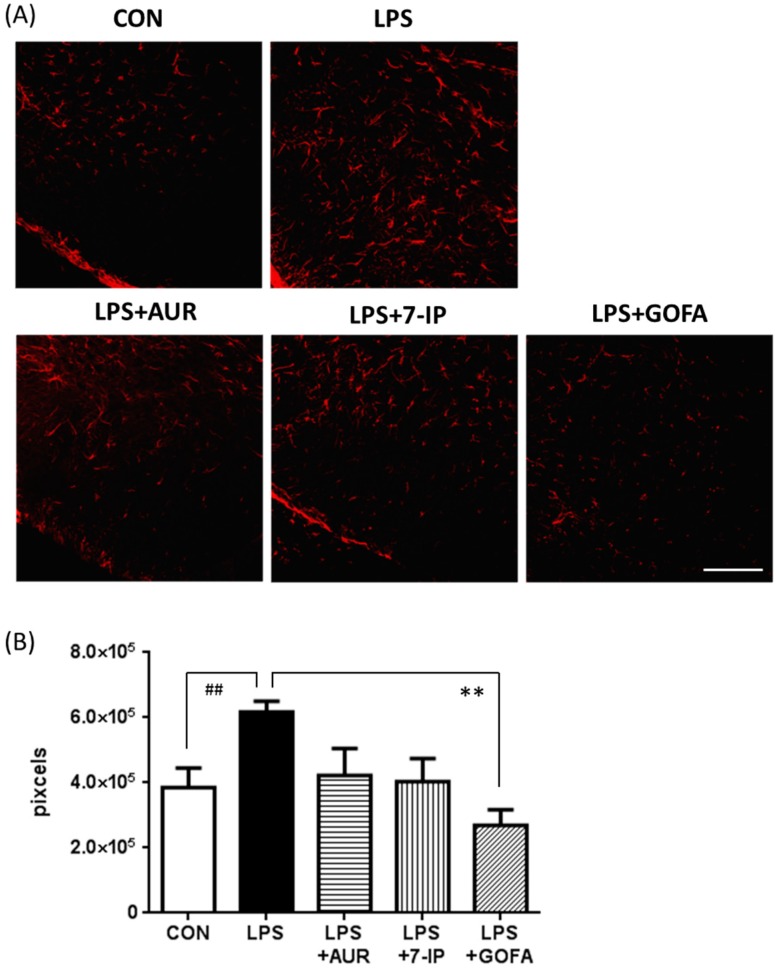
Effects of AUR, 7-IP, and GOFA on the LPS-induced activation of astrocytes in the substantia nigra. (**A**) Representative confocal micrographs of glial fibrillary acidic protein (GFAP)-positive cells on day 22 in the indicated groups. Scale bar = 100 μm; (**B**) Total GFAP-positive cells signal densities. Values are the means ± SEM. Significant differences were observed between CON and LPS (^##^ *p* < 0.01) and between LPS and sample treatment groups (** *p* < 0.01).

Astrocytes release inflammatory mediators [27], and are activated by the administration of LPS, resulting in the so-called “reactive gliosis” [28]. We stained activated astrocytes with an antibody against glial fibrillary acidic protein (GFAP). As shown in Figure 4A, GFAP-positive astrocytes in the SN were sparse in the CON group, but dense in the LPS group. Figure 4B shows that the area of these cells in the LPS group was approximately 1.6-fold that in the CON group (^##^ *p* < 0.01). GFAP immunoreactivity in the LPS + GOFA group was approximately 43.5% that in the LPS group, and was markedly lower than that in the CON group (Figure 4A,B; ** *p* < 0.01 vs. the LPS group). In the LPS + AUR and LPS + 7-IP groups, activated astrocyte levels decreased to approximately 68.4% and 65.3% that in the LPS group, respectively, which were similar to that in the CON group (Figure 4A,B). Nevertheless, no significant differences were observed among the LPS, LPS + AUR, and LPS + 7-IP groups. These results indicate that GOFA has the ability to suppress LPS-induced astroglial hyperactivation in the SN, while AUR and 7-IP exhibit weak abilities.

### 2.4. Effects of AUR, 7-IP, and GOFA on LPS-Induced Neuronal Cell Loss in the SN

**Figure 5:**
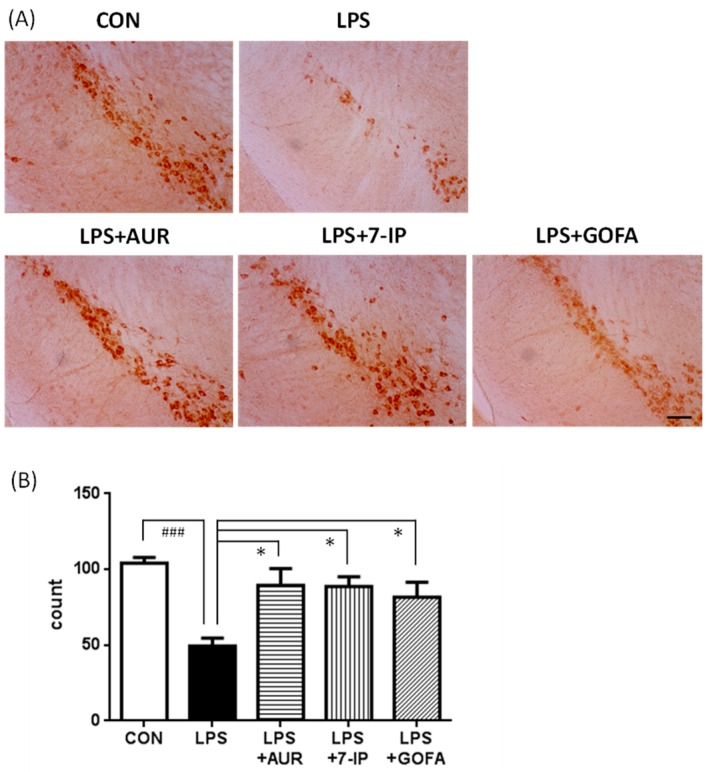
Effects of AUR, 7-IP, and GOFA on LPS-induced neuronal cell loss in the substantia nigra. (**A**) Representative micrographs of tyrosine hydroxylase (TH)-positive neuronal cells on day 22 in the indicated groups. Scale bar = 100 μm; (**B**) Total intact cell number of TH-positive neuronal cells. Values are the means ± SEM. Significant differences were observed between CON and LPS (^###^ *p* < 0.001) and between LPS and sample treatment groups (* *p* < 0.05).

In PD, dopaminergic neurons are particularly vulnerable to inflammation [29]. Therefore, we stained dopaminergic neurons in the SN pars compacta with an anti-tyrosine hydroxylase (TH) antibody (Figure 5A), and intact cells were manually counted (Figure 5B). The number of TH-positive neuronal cells in the LPS group was approximately half of the CON group (^###^ *p* < 0.001). The numbers of TH-positive neuronal cells in the LPS + AUR, LPS + 7-IP, and LPS + GOFA groups were 2.3-fold (* *p* < 0.05), 2.3-fold (* *p* < 0.05), and 1.9-fold (* *p* < 0.05) that in the LPS group, respectively.

**Figure 6:**
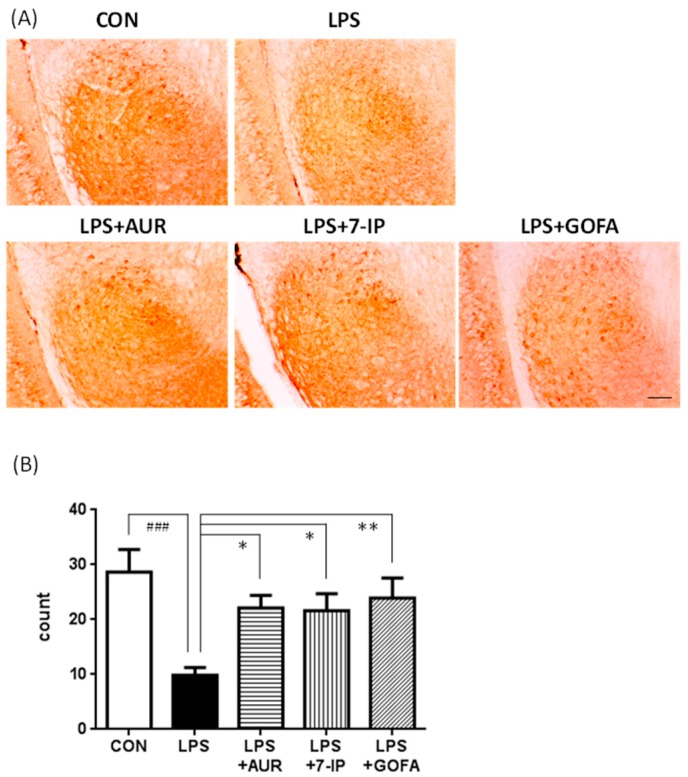
Effects of AUR, 7-IP, and GOFA on LPS-induced neuronal cell loss in the substantia nigra. (**A**) Representative micrographs of GAD67-positive neuronal cells on day 22 in the indicated groups. Scale bar = 100 μm; (**B**) Total intact cell number of GAD67-positive neuronal cells. Values are the means ± SEM. Significant differences were observed between CON and LPS (^###^ *p* < 0.001) and between LPS and sample treatment groups (* *p* < 0.05, ** *p* < 0.01).

Furthermore, it is well known that the pars reticulata of the SN receives γ aminobutyric acid (GABA)-ergic input from the neostriatum, which in turn, there are outputs to thalamic nuclei from the SN. We thus stained GABAergic neurons in the SN pars reticulata with an anti-glutamic acid decarboxylase (GAD) 67 antibody (Figure 6A), and intact cells were manually counted (Figure 6B). The number of GAD67-positive neuronal cells in the CON group was approximately 2.9-fold that in LPS the group (^###^ *p* < 0.001). The numbers of GAD67-positive neuronal cells in the LPS + AUR, LPS + 7-IP, and LPS + GOFA groups were 2.2-fold (* *p* < 0.05), 2.2-fold (* *p* < 0.05), and 2.4-fold (** *p* < 0.01) that in the LPS group, respectively. These results indicate that AUR and its analogs (7-IP and GOFA) exhibit neuroprotective abilities in the SN of PD-like model mice.

## 3. Discussion

PD is the second most common neurodegenerative disease and its pathological characteristics are the slow and progressive degeneration of dopaminergic neurons in the SN and associated motor dysfunction [30,31]. Although the precise etiology of PD remains unclear, aging, oxidative damage, and neuroinflammation have been shown to play crucial roles in its pathogenesis [32]. Neuroinflammation, in particular microglial activation, has recently been attracting a great deal of attention as the key player in PD because activated microglia have been observed to enclose the lost dopaminergic neurons in the brains of PD patients and PD animal models [12,13]. When taken together with our findings that AUR suppressed the activation of microglia in ischemic brains [9,10] and inflammatory brains [11], we were prompted to investigate the effects of AUR on the brains of LPS-induced PD model mice. In the present study, we observed that AUR suppressed microglial activation and subsequent dopaminergic neuronal cell death in the SN of the brains of LPS-induced PD model mice, as expected. We also examined the effects of other prenyloxyphenylpropanoids, such as 7-IP containing a coumarin structure with an isopentenyl chain and GOFA containing a geranyloxyl chain on cinnamic acid. The results obtained showed that all three compounds tested exhibited the ability to suppress microglial activation (Figure 3) and consequently inhibit neuronal cell death in SN of PD model mice (Figure 5 and Figure 6). In our preliminary experiment using inflamed model mice induced by an intraperitoneal injection of LPS, AUR, 7IP, and GOFA exerted suppressive effects on microglial hyperactivation in the hippocampal region of their brains (unpublished data). Consequently, this is the first study to show that 7-IP and GOFA exert anti-inflammatory effects in the brain and also that some natural prenyloxyphenylpropanoids including AUR, 7-IP, and GOFA may serve as a therapeutic tool for PD.

When Epifano et al. [7] investigated the neuroprotective effects of 8 selected prenyloxyphenylpropanoids on NMDA-induced toxicity in vitro, compounds containing a coumarin structure such as AUR and 7-IP were effective as neuroprotective agents, and the neuroprotective ability of 7-IP was greater than that of AUR. On the other hand, the other 6 compounds without a coumarin structure, such as GOFA, did not exhibit any neuroprotective activity. These results suggest that (1) the coumarin ring is crucial for neurotrophic effects against excitotoxicity; and (2) the presence of an isoprenoloxy chain on the coumarin structure is more effective than a geranyloxyl chain. Toxicity induced by glutamate was accompanied by an increase in the release of d-aspartate, enhanced ROS and nitric oxide (NO) production, and an impaired mitochondrial membrane potential [33]. Coumarins have been reported to exert neuroprotective effects as radical scavengers [34]. The neuroprotective abilities of prenyloxyphenylpropanoids against NMDA excitotoxicity (probably against ROS) may be mediated by the coumarin structure.

Our present results showing that AUR, 7-IP, and GOFA exert neuroprotective effects against LPS-induced inflammation suggest that the mechanisms responsible for the anti-inflammatory effects of prenyloxyphenylpropanoids may differ from those underlying their anti-oxidant effects (not dependent on the coumarin structure). Only one dose of these three compounds was examined here (83.8 μmol/kg/day); therefore, a dose-response investigation will be necessary in the future in order to clarify which compounds are more effective and elucidate the type of relationship that exists between their structures and activities.

Other than microglia, astrocytes are important cells in the pathogenesis of PD due to their inflammatory and anti-inflammatory actions [35]. Hyperactivated astrocytes may release chemokines and cytokines including tumor necrosis factor (TNF)-α and interleukin (IL)-1β [16,29,35], which further induce microglial and astroglial activation. Figure 4 shows that the LPS injection into the SN induced the activation of not only microglia, but also astrocytes as previously reported [20]. Figure 4 also shows that GOFA significantly suppressed astroglial activation, while AUR and 7-IP slightly suppressed it. These results support the suggestion that the anti-inflammatory effects of prenyloxyphenylpropanoids are not dependent on the coumarin structure.

In contrast, the astrocytes are known to increase neurotrophic factor synthesis such as brain-derived neurotrophic factor (BDNF) and glial cell line-derived neurotrophic factor (GDNF) [36]. Therefore, we investigated whether the treatments with AUR, 7-IP, and GOFA affected the expression levels of GDNF in this experiment. The results obtained showed that these compounds did not cause significant changes in its expression (data not shown). A time-dependent study is warranted in the future in order to clarify whether AUR, 7-IP, and GOFA exert neuroprotective effects through the up-regulation of neurotrophic factors including BDNF and/or GDNF.

When we evaluated the behavior of LPS-treated mice by rotarod test (a performance test based on a rotating rod with forced motor activity) in this experiment, their behavior were normal (data not shown), probably because LPS treatment in the present experimental condition might not induce motor system dysfunction yet in the SN and striatum dopaminergic pathway system. Consequently, we could not yet evaluate the effects of AUR, 7-IP, and GOFA on motor dysfunction behavior. We intend to evaluate the effects of these compounds on behavioral performance using other experimental condition with other methods such as open field test.

Nevertheless, Okuyama et al. recently reported the ability of AUR to penetrate the brain [10]. The permeability of 7-IP and GOFA through the blood-brain-barrier (BBB) will be examined in future studies.

## 4. Materials and Methods

### 4.1. Materials

AUR was kindly gifted from USHIO Chemix, Co., Ltd. (Omaezaki, Japan) and its purity confirmed by NMR and HPLC was ≥99.7%. 7-IP and GOFA were synthesized as previously described [37] and their purities (≥98.7%) confirmed using NMR. These three compounds were dissolved in DMSO/polyethylene glycol 300 (1:9). LPS (from Salmonella enteric serotype typhimurium) was purchased from Sigma-Aldrich (St. Louis, MO, USA), and dissolved in saline.

### 4.2. Animals

Nine-week-old male C57BL/6 mice were obtained from Japan SLC (Hamamatsu, Japan). Mice were kept at 23 ± 1 °C and a 12-h light/dark cycle (light on 8:00–20:00), and had free access to tap water and food during the experimental period. All animal experiments were carried out in accordance with the Guidelines for Animal Experimentation prepared by the Animal Care and Use Committee of Matsuyama University (approved on 2 September 2009, Protocol No. 09-002).

### 4.3. Intranigral Injection of LPS

The procedure for the single intranigral injection of LPS was performed as described previously [16] with slight modifications. Mice were anesthetized with pentobarbital and subjected to the intranigral injection using a stereotaxic device (IMPACT-1000B; Muromachi Kikai Co., Ltd., Tokyo, Japan), and a linear skin incision was made over the bregma. Two 1-mm burr holes were drilled in the skull 2.8-mm posterior and 1.3-mm lateral to the bregma on both sides using a hand-held driller. One microliter of LPS solution (the dose of LPS was 3 μg in PBS) was injected 4.5 mm below the surface of the skull using a 10-μL Hamilton syringe for each hemisphere; therefore, 6 μg/2 μL of a sample was injected into each mouse (both hemispheres). Control animals underwent the same surgical procedure with a PBS intranigral injection. After surgery, all mice were placed in a recovery cage under a heat lamp.

### 4.4. Administration of AUR, 7-IP, or GOFA

Mice were divided randomly into five experimental groups (*n* = 9–10): a PBS-injected control group (CON), LPS-injected group (LPS), AUR (25 mg/kg/day)-treated group (LPS + AUR), 7-IP (19.3 mg/kg/day)-treated group (LPS + 7-IP) and GOFA (27.7 mg/kg/day)-treated group (LPS + GOFA). Mice in the 3 groups (LPS + AUR, LPS + 7-IP, and LPS + GOFA) were subcutaneously administered each sample solution (500 μL/kg) to achieve 83.8 μmol/kg/day, once a day for 21 days after the LPS intranigral injection of the day 1. Mice in the CON and LPS groups received vehicle solution subcutaneously for 21 days.

### 4.5. Immunohistochemistry for Optical Microscopy

On day 22, mice were transcardially perfused with ice-cold PBS, and brains were removed and postfixed with 4% paraformaldehyde. 30 μm-thick coronal sections of frozen brains were cut using a cryostat (CM3050S; Leica Microsystems, Heidelberger, Germany). Brain sections were immunostained using a rabbit polyclonal antibody against IBA1 (Wako, Osaka, Japan) to stain microglia/macrophages, a rabbit polyclonal antibody against TH (Cell signaling, Woburn, MA, USA) to stain dopaminergic neurons, and mouse monoclonal antibody against GAD67 (Abcam, Cambridge, UK) to stain GABAergic neurons. The secondary antibody used was an EnVision-plus system HRP-labeled polymer (anti-rabbit or anti-mouse; Dako, Glostrup, Denmark). Immunoreactivity was developed and visualized by DAB substrate (SK-4100; Vector Laboratories, Burlingame, CA, USA). Regarding the quantification of IBA1-positive signals, ImageJ software (NIH, Bethesda, MD, USA) was used as previously described [10], and TH and GAD67-positive signals were manually counted.

### 4.6. Immunohistochemistry for Confocal Fluorescence Microscopy

30 μm-thick coronal sections were incubated with the primary antibody of mouse anti-GFAP (Sigma-Aldrich) as a marker for activated astrocytes, and the secondary antibody was Alexa Fluor 568 goat anti-mouse IgG (H + L) (Invitrogen, Carlsbad, CA, USA). Mounting medium (Vectashield; Vector Laboratories) was used, and images of the SN were captured with a confocal fluorescence microscopy system (LSM510; Zeiss, Oberkochen, Germany). GFAP-positive signals were quantified using ImageJ software (NIH), similar to IBA1 staining.

### 4.7. Statistical Analysis

Data for individual groups are expressed as means ± SEM. Data were analyzed using a *t*-test between the CON and LPS groups, and a one-factor ANOVA followed by Dunnett’s Multiple Comparison Test between LPS and each sample-treated group (Prism 6; GraphPad Software, La Jolla, CA, USA). A value of *p* < 0.05 was considered to be significant.

## 5. Conclusions

The results of the present study showed that AUR, 7-IP, and GOFA suppressed LPS-induced inflammatory responses in the brain and, as a consequence, neuronal cell death. Since inflammation is associated with not only PD, but also other neurodegenerative diseases such as Alzheimer’s disease, natural prenyloxyphenylpropanoids containing AUR, 7IP, and GOFA may be useful in the treatment of neurodegenerative diseases.
